# Hydatid Disease Simulating Acute Abdomen: A Case Report and Brief Review of the Literature

**DOI:** 10.1155/2012/387102

**Published:** 2012-09-20

**Authors:** Georgios Lianos, Georgios Baltogiannis, Avrilios Lazaros, Konstantinos Vlachos

**Affiliations:** Department of Surgery, University Hospital of Ioannina, St. Niarchou Avenue, 45110 Ioannina, Greece

## Abstract

*Introduction*. Hydatid disease is caused by the tapeworm *Echinococcus granulosus* and is still a matter of public health in many regions of the world, where it is an endemic parasitic disease. Although the liver is the most involved organ, hydatidosis can be found anywhere in the human body. Rare forms of location may lead to diagnostic and therapeutic dilemmas. *Case Report*. Herein we report a rare case of acute abdominal pain and progressively increasing abdominal distension due to abdominal and multiple splenic echinococcosis in a 72-year-old Caucasian male. We also provide a brief review of the literature. *Conclusion*. Although hydatid disease is found most often in the liver and lungs, rarely any organ of the body can be involved by this zoonosis. Though rare, the possibility of unusual location of echinococcosis must always be considered by the operating surgeon, when dealing with diffuse abdominal pain in endemic areas, because any misinterpretation may result in unfavorable outcomes.

## 1. Introduction

Echinococcosis is a zoonosis that continues to be a major health problem in many countries. It is endemic in several countries in South America, the Middle and Far East, and around the Mediterranean Sea [1, 2]. Hydatid disease is caused by the larval form of *Taenia echinococcus*, which lives in the gut of dog, wild canines, and carnivorous animals that represent the definitive hosts [3]. Humans become the accidentally intermediate hosts by ingesting Taenia eggs. Then the slowly growing echinococcal cysts can achieve a volume of several liters and contain many thousands of protoscolices. With time daughter cysts can be formed. The liver is the organ most involved by echinococcosis (65%–70%) followed by the lungs (25%). Less frequently hydatidosis involves the spleen, kidneys, peritoneum, brain, heart, and bones [4]. To our knowledge, only few reports on abdominal hydatid disease simulating acute abdomen have been published to date. We share our experience in successfully treatment of such a unique case and we provide a brief review of the literature in this issue.

## 2. Case Report

**Figure 1:**
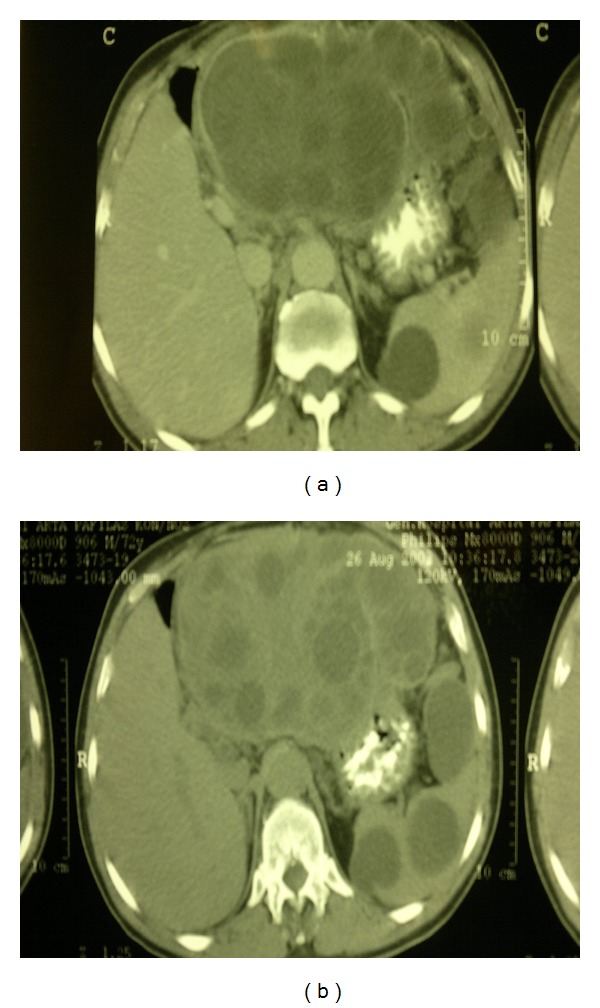
CT showed a large echinococcal cyst with daughter cysts in the left lobe of the liver, splenic echinococcosis and a large echinococcal cyst in the left hypochondrium causing a huge pressure to the stomach.

A 72-year-old Caucasian male was admitted to the emergency department of the University Hospital of Ioannina due to diffuse abdominal pain, nausea and progressively increasing abdominal distension of ten-day duration. His medical history was free and no recent trauma was reported. Clinical examination revealed pyrexia of 38.5°C, tachycardia of 110 per minute, and normal blood pressure. Upon physical examination, a grossly distended and diffusely tender abdomen was revealed. On auscultation abdominal sounds were present. Rectal examination showed an empty rectum. The emergent laboratory tests revealed as follow: WBC 14700 mm^3^, haemoglobin at 10.5 g/dL, c-reactive protein was at 148 mg/l, creatinine, electrolytes, and liver function tests were normal. The examination of urine was also normal. The abdominal plan X-ray was normal and an urgent abdominal CT was arranged. The CT revealed a huge hepatic hydatid cyst (measuring 14.5 cm) with daughter cysts in the left lobe of the liver and multiple splenic hydatidosis. Additionally, a large echinococcal cyst was revealed at the left hypochondrium attached to the anterior wall of the stomach causing a huge pressure (Figures 1(a) and 1(b)). Hydatid cysts were also revealed in the transverse mesocolon and in pelvis. Laparotomy was performed the day after the admission due to progressively increasing abdominal distension and to diffuse abdominal pain. Intraoperative findings confirmed the diagnosis of abdominal and splenic hydatidosis. The patient was treated with deroofing and omentoplasty for the large hepatic echinococcal cyst, splenectomy for the splenic cysts, and removal of the rest echinococcal cysts. The postoperative period was uneventful and the patient was discharged 15 days later, with the advice to receive albendazole for three months postoperatively.

## 3. Discussion

Echinococcosis produced by *Echinococcus granulosus* still represents an important medical problem in many countries, Greece included. Hydatid disease remains a challenging surgical condition worldwide. Even though many infections are acquired in childhood, most cases of liver and lung involvement become symptomatic in adult patients because of the slowly growing nature of the cysts. Echinococcosis in extrahepatic sites is usually asymptomatic unless the cyst causes symptoms due to pressure, as in our case, or ruptures to the peritoneal cavity [5, 6]. The symptoms of hydatid disease are determined by the size, the site, and the condition of the cysts. Echinococcal cysts at unusual locations many times pose diagnostic dilemmas, and the diagnosis sometimes is made intraoperatively [7]. We found in literature some case reports presenting unusual location of hydatidosis, but it seems that only few cases of abdominal echinococcosis simulating acute abdomen have been published to date [8–13]. It is also reported by Ozalp et al. a case of peritoneal hydatidosis presenting with ileus. Exploratory laparotomy revealed multiple peritoneal hydatid disease and an echinococcal cyst in the right lobe of the liver [14]. Karavias et al. report that intraperitoneal hydatid disease accounts for 12% of all abdominal echinococcosis and that the cyst development and growing are secondary to spontaneous or iatrogenic rupture or microrupture of splenic or hepatic echinococcal cysts. The intraperitoneal cyst can be located anywhere in the peritoneum [15]. On the other hand, splenic echinococcal involvement is relatively rare and its incidence has been reported from 0.9% to 8%. It is also referred that splenic echinococcal cysts are usually solitary. Simultaneous hepatic, multiple splenic, and peritoneal hydatid disease presenting as acute abdomen, as our case, is believed to be a unique clinical case [16, 17]. It is confirmed that echinococcosis can be found in every organ of the body and that must be considered in the differential diagnosis of acute abdomen, especially in endemic areas. Additional measures, such as the education of farmers and general public, are essential in the control of this zoonotic infection in animals and humans [18].

## 4. Conclusion

In conclusion, it seems that the ideal treatment of hydatid extrahepatic disease is total excision (total cystectomy) with no organ resection, if it is feasible. Surgical intervention is required for symptomatic and exceptionally large peritoneal cysts. The diagnosis of uncommon locations of echinococcosis can be very difficult and requires a high suspicion and a lot of awareness on the part of the surgeon. The most important factor in diagnosing hydatid disease in unusual locations is the awareness of its possibility. Hydatid disease should be considered in the differential diagnosis of acute abdomen in endemic areas.
